# Chinese Women’s Acceptance and Uptake of Referral after Screening for Perinatal Depression

**DOI:** 10.3390/ijerph17228686

**Published:** 2020-11-23

**Authors:** Wenjie Gong, Xin Jin, Kar Keung Cheng, Eric D. Caine, Richard Lehman, Dong (Roman) Xu

**Affiliations:** 1 Xiangya School of Public Health, Central South University, 110 Xiangya Road, Changsha 410078, China; gongwenjie@csu.edu.cn (W.G.); jinx0423@csu.edu.cn (X.J.); 2 Institute and of Applied Health Research, University of Birmingham, Birmingham B15 2TT, UK; K.K.Cheng@bham.ac.uk (K.K.C.); R.Lehman@bham.ac.uk (R.L.); 3 Department of Psychiatry, University of Rochester, 300 Crittenden Blvd, Rochester, NY 14642, USA; eric_caine@urmc.rochester.edu; 4 Global Health and Health System, ACACIA Labs and Department of Health Management, School of Health Management, Southern Medical University, 1023 South Shatai Road, Guangzhou 510515, China

**Keywords:** perinatal depression, routine screening, referral, preferences for care, mental health

## Abstract

China recently issued a national plan on perinatal depression (PND) screening. Previous studies elsewhere suggested that uptake of referral after screening for PND is suboptimal, but little is known in China. In this cohort study including 1126 women in Hunan, we identified women at a high risk of PND using the Edinburgh Postpartum Depression Scale (EPDS) over multiple time points. We texted them and offered free consultations with a psychiatrist/psychologist. Among 248 screen-positive women, only three expressed interest and one attended the appointment. We surveyed the women about their reasons for declining referrals and preferred means of care. Of the 161 respondents, 128 (79.5%) indicated that they could cope with the condition without professional assistance and 142 (88.2%) chose their families as the preferred source of help. Only 15 (9.3%) chose professionals as their first option. Implementing a referral policy for screen-positive women would mean approximately one-third of women who gave birth in China would be eligible. Our result argues against referring all screen-positive women for professional services at this time. Interventions should instead build upon the tradition of family support in a more engaged response. These considerations are relevant for the implementation of national screening for PND in China.

## 1. Introduction

On 11 September, 2020, the National Health Commission of China (NHC) issued a work plan to introduce screening for depression during pregnancy and childbirth to routine pregnancy tests and postpartum visits [1]. Depression during pregnancy and childbirth, also known as perinatal depression (PND), is a public health problem that encompasses clinically significant depressive conditions occurring during pregnancy or within the first 4–6 weeks after delivery. The reported prevalence of PND in low- and middle-income countries is 15.6% antenatally and 19.8% postnatally [2]. In mainland China, the prevalence was estimated to be 16.3% [3]. Negative consequences for the mother and, in turn, for her infant and family may include impaired mother–infant attachment with resulting impact on healthy child development, suicidal thinking, and, in the extreme, suicide [4,5,6]. Health agencies in the EU, the U.S., and other developed countries recommend regular screening of pregnant and post-partum women to detect symptoms suggestive of PND [7,8,9,10,11,12].

PND screening alone, although potentially increasing the identification of symptomatic women, is not sufficient for improving health outcomes if it does not lead to timely referrals and effective interventions. A previous systematic review found that only 22% (13.8–33.0%) of women who screened positive for PND received at least one mental health visit when referred to care. Higher acceptance rates followed additional interventions, including resource provision to women, perinatal care provider training, on-site assessment, and access to mental health consultation for perinatal care providers. All 17 studies included in this review were conducted in economically developed countries, with 13 from the United States [13]. We updated this review and found 41 studies related to the acceptance of referral after PND screening [14]. Only two were not conducted in high-income countries; they were from South Africa and Turkey. We could not find any study from mainland China. 

In 2019, there were approximately 14.65 million births in China [15], with an estimated 5.06 million (34.53%) women who would have been screen-positive for PND if the Patient Health Questionnaire-9 (PHQ-9) was used as a screening instrument (which was recommended in the work plan) [16]. Whether the large number of women affected would accept the results of the screening, whether they would be referred, and whether they would accept help will directly determine the effectiveness of the previously mentioned work plan. A large majority of the women follow Chinese customary practices during the postpartum period, especially the tradition of “doing the month”—a socially prescribed period of rest and restraint when women are confined to their homes and strictly limited in their activities, while their mothers or mothers-in-law take charge of their wellbeing and that of the newborn [17,18]. This practice is deeply entrenched in Chinese culture and is different in many ways from Western cultures [17,18,19] and is likely to affect the attitudes and responses to referral for PND. In this study, we sought to assess whether women who screen positive for PND during or after pregnancy would accept an offer of a mental health consultation. We also explored their preferences for support from the health service and other sources. 

## 2. Materials and Methods 

### 2.1. Participants

This study was part of a cohort study that examined the incidence, detection, trajectory, and correlates of depression among women from the first trimester of pregnancy through 6 weeks postpartum, with a maximum of seven assessments [20]. The study received approval from the institutional review board of the Institute of Clinical Pharmacology of Central South University (No.CTXY-140002-2). During 6 consecutive months, we approached every pregnant woman, gestation ≤ 12 weeks, in two collaborating maternal and child hospitals in Hunan Province, PRC: the major provincial teaching hospital located in the capital, Changsha (a city of approximately 7.9 million), and a second county-level institution in Yiyang (population approximately 4.3 million). Both hospitals draw people from urban and surrounding rural areas. All participants provided informed consent. In total, 1126 women were recruited from September 2016 to February 2017.

### 2.2. Procedures and Outcome 

Trained research staff collected basic socioeconomic and demographic information at the time of participant recruitment. 

**Figure 1:**
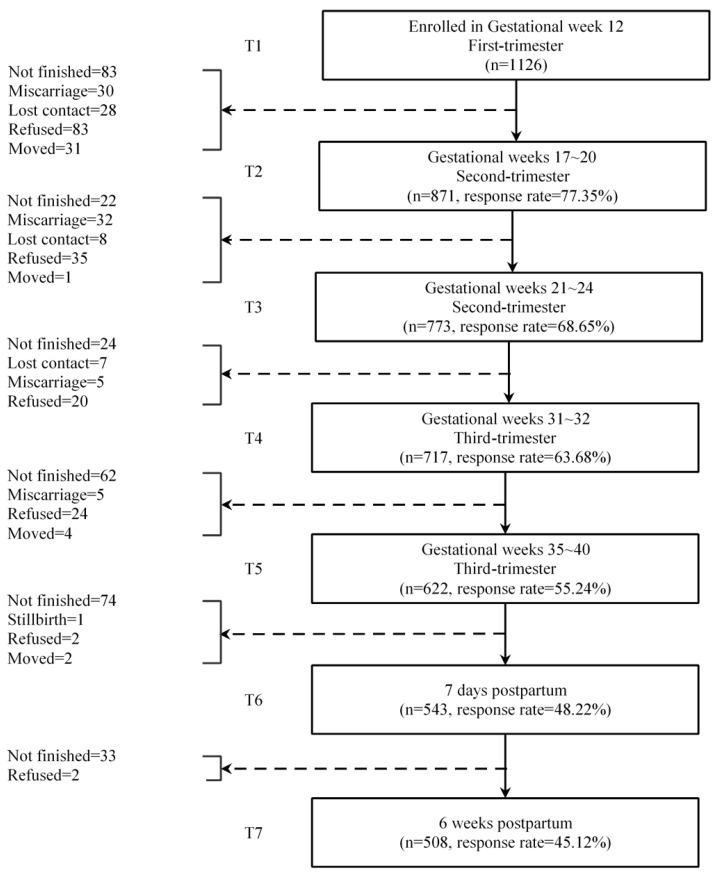
Flow chart of the cohort study.

Identifying individuals reporting symptoms consistent with what we labeled as having “high risk” of perinatal depression was the primary objective of our study. We screened participants with the Edinburgh Postpartum Depression Scale (EPDS), which was administered prenatally at (approximately) weeks 12, 20, 24, 32, and 38; within one week after delivery; and at 42 days postpartum when women returned for a routine follow-up appointment (Figure 1). For this study, we set the following more stringent criteria for inclusion as high risk compared to the typical cut-off score of 10 or more: (1) women scoring 13 or more at least twice (consecutively or non-consecutively) or (2) a positive response to EPDS item 10 on any survey, indicating suicidal thoughts or (3) both. 

We sent each screen-positive respondent an alert message by text (all participants consented to contact) to inform them of their results if the pregnant women met the criteria for high risk and offered a consultation with psychiatrists or psychologists. The alert message stated: “Thank you for participating in our screening project. We found that you have a high score for the screening, which indicates a heightened risk for depression. You do not need to panic as this high score may be due to changes in hormones and environment. Timely consultation with health specialists may help you obtain expert advice on improving your situation. If you need any assistance in a referral or more relevant information, please contact us at [phone number].” If individuals ignored our initial alert, we resent it a week later. 

For those having no response after two alerts, we sent a text message connecting them to an anonymous online survey that we developed to understand their reasons for not seeking referrals. Questions included: (1) What did you feel when you received our message (i.e., surprised, suspicious, or accepting)? (2) What prevents you from asking for further information (i.e., stigma, cost, or medication side effect)? (3) Would you visit a psychiatrist/psychologist if we made an appointment for you? If not, why? (4) Would you like to use a smart-phone therapeutic application to treat depression? Why not? (5) What resource of help would you want to get to deal with the depression (i.e., family, specialist, or social worker)? In this survey, questions 2–5 asked respondents to rank their reasons/resources by placing options in order of preference. The average ranking score (a continuous variable starting from 0) was calculated for each option, with a higher score indicative of a stronger overall preference for a specific choice [21]. The last survey link was sent in August 2017.

### 2.3. Analysis

We conducted descriptive analyses of the data, which are presented as tables and figures. When necessary, appropriate statistical tests were performed. We asked people to sort reasons in order of importance for questions 2–5. We calculated a ranking score for those items—a higher score indicating the weighted higher frequency of selecting this item by the respondents (Σ frequency by weight)/number of subjects who responded with the weight determined by where the respondents order this item in terms of its importance [22].

## 3. Results

### 3.1. Profile of Women Having a High Risk of Depression

Of the 1126 subjects recruited, 508 (45.1%) were assessed with the EPDS the maximum of seven times and an additional 257 (765, 65.3%) participants completed at least five EPDS assessments [20].

From among the entry sample, 248 women (22.0%) were identified as high risk. Of these, 63 scored 13 or more on the EPDS at least twice, 111 responded to EPDS item 10 indicating thoughts of self-harm on at least a single survey, and 74 met both criteria. Twelve reported being diagnosed with major depression before the start of the study. Table 1 shows that high-risk women had a median age of 29 years; most lived with their families, were employed, and were highly educated. With few exceptions, demographic characteristics did not differentiate screen-positive women from their asymptomatic peers. However, fewer of the screen-positive respondents were satisfied with their spousal relationship, while a higher percentage of screen-positive respondents were somewhat satisfied. Although slightly fewer screen-positive women said that they would “do the month,” the vast majority of both groups affirmed traditional Chinese postpartum family care. Among positive responders, there was a small though statistically significant tendency for families to favor boys over girls.

### 3.2. Responses to Referral

Among the 248 high-risk women to whom we sent the initial alert message, only 3 (3/248, 1.2%) contacted us for further information. Each attended the provincial hospital in Changsha. Although we helped arrange an appointment with psychiatrists to coincide with their routine maternal care visit and offered to cover their outpatient expenses, only one (0.4%, 1/248) went to the appointment.

**Figure 2:**
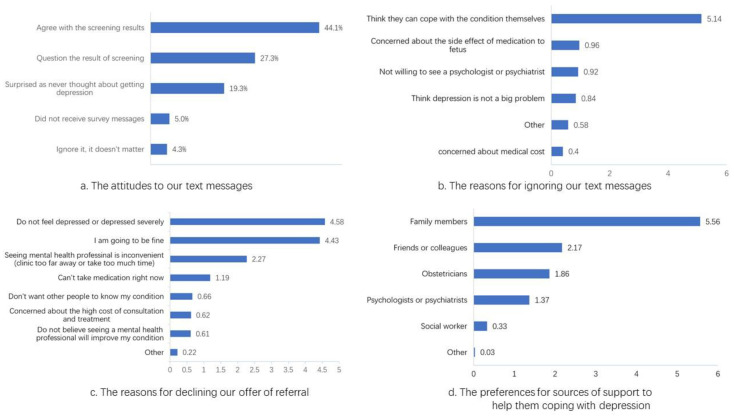
The responses of screen-positive women using ranking scores to indicate the importance of the items among our survey respondents.

Of the 245 women who did not respond to alerts, 161 (64.7%), divided nearly equally between the major provincial maternal-child hospital (*n* = 80) and the county hospital (*n* = 81), completed the online survey regarding their reasons for not accepting the referral. Among them, 71 (44.1%) agreed with the results about being depressed, while 44 (27.3%) contested the accuracy of the results. Among the latter, 32 (19.3% of the online responders) had never considered the possibility of developing some type of depressive condition (Figure 2a).

### 3.3. Reasons for not Accepting Referrals

The majority (128/161; 79.5%) of the respondents affirmed that they did not ask for further information because “I can cope with the condition myself.” Of the 128, 89 (84.0%) selected this option as their primary reason for not seeking help. After weighted sorting, the importance of each reason is shown in Figure 2b. Among the other reasons for not seeking assistance, 5 of 19 respondents listed, “I am fine/good,” and three mentioned time conflict. Only one person explained that she had been treated in the psychology department already.

Of the 161 women, 106 (65.8%) said they would not accept our offer for arranging appointments with mental health specialists; 68 (64.2%) agreed with the statement, “I feel that I am not depressed or my depression is not severe.” Another 63 (59.4%) indicated: “I am going to be fine.” Only 13 (12.3%) expressed concerns about the cost of diagnosis and treatment, and only three ranked this choice as most important. All the reasons sorted by importance are shown in Figure 2c. It is notable, however, that 134 (83.2%) of the 161 respondents expressed interest in trying smartphone application (app)-based therapies. Of the 27 (16.77%) people who did not express an interest, most indicated that they believed that they were not depressed or they could care for themselves.

### 3.4. Preference for Care

When asked about which sources of help they might seek for their depression, 151 (93.8%) women indicated their families, and 142 listed their families as the most important source of support, followed by friends and colleagues (*n* = 77; 47.8%). Few preferred services from professionals (*n* = 15; obstetricians for nine and psychologists or psychiatrists for six), and none chose social workers. All the sources sorted by preference are shown in Figure 2d.

## 4. Discussion

We found that symptoms consistent with PND were common among the Chinese women who participated in this study. Using a generally conservative threshold for defining positive screening with EPDS, we found that 248 of 1126 women (22.0%) in our sample were screen-positive, reflecting either a repeated score ≥13 or a positive self-harm score. If we had used ≥10 as our screening cut-off, as recommended by the validity study of the Chinese version of the EPDS [23,24], nearly 40% of our participants would have screened positive at least once, similar to other PND studies in China [25,26]. Even with more stringent criteria, it was readily evident that participants had little interest in pursuing further medical evaluation or treatment of their conditions: only 3 out of 248 women (1.21%) who screened positive for depression in our study expressed interest in obtaining a mental health evaluation, and only 1 (0.40%) of 248 availed herself of this service.

If we were to implement a mental health referral policy for screen-positive women during the perinatal period using PHQ-9, approximately one-third of women who gave birth in China would have been eligible, as we calculated in the introduction [16]. However, the lack of services and clinical expertise precludes such a policy. Few obstetrician-gynecologists or primary care providers in China have dealt with PND in their practices [27]. With nearly all of China’s psychiatrists (29,924 in 2017) based in large psychiatric hospitals with overcrowded outpatient clinics [28], the potential for symptomatic women to receive careful evaluations and appropriate treatment is extremely limited.

The results of our study demonstrated that the women responding to our survey did not regard their mental distress as a medical problem. Having resources available was a moot issue, given the lack of demand. In the systematic review of 17 studies from Western cultures, the highest referral acceptance rate was 33.0%, the lowest was 13.8% without any intervention [29,30]. Our finding was much lower than prior studies—only 0.4%.

After we uncovered this low uptake of referral, we published a systematic review on the issue of uptake of referral [14]. In that review, we found that the top reasons were lack of time and perception that mood had improved [31,32,33,34,35,36,37]. Other reasons included cost concerns, transportation problems, and the perception of the nature of PND [31,33,38]. Stigma associated with psychiatric treatments deterred the uptake of referral as well [38,39]. Furthermore, the perception that it is normal to have some depression in the puerperium prevented the need for further health care [40,41]. Women’s preferences for the type of service offered also influenced the level of acceptance. The literature suggests that many women prefer home visitation to specialized services and that some women tended to use their own resources instead of professional care [32,33,42,43]. We are currently conducting qualitative research to explore in depth the causes behind this observation in China.

Cultural differences may partially explain the extraordinarily low uptake rate for referral for mental health services among Chinese women. Traditional Chinese culture emphasizes self-restraint and introspection, requiring people to rely on their own strength to solve internal conflicts [44]. Many studies have found that cultural barriers, consisting of credibility of treatment, recognition of need, and fear of loss of reputation are reasons for the low rate of seeking of mental health care among Chinese immigrants [45,46,47,48]. Similar to the stigmatization of mental disorders in many societies, this cultural attitude would reduce uptake of referral and would need to be addressed by health education in the long term. Cost was a less important factor influencing the low uptake rate. This is different from many studies in Western countries [33,38,49]. While the relatively low cost of psychiatric care in China may be one reason, it may also indicate that practical barriers are not really important until cultural barriers are overcome.

It is debatable whether the screening-based referral constitutes the most cost-effective approach for tackling this mental health challenge in China. In our related longitudinal study, we found that even without any medical treatment, many women may have recovered from their depression. Our web-based survey indicated a large proportion of the high-risk women′s EPDS scores dropped below 12.5, and more than half of them were below 9.5 at 6 months and one year following the delivery (Appendix A). The 50% attrition rate of the cohort was relatively high, although no difference was shown between at-risk and other women. These findings taken one year postpartum suggest spontaneous resolution of symptoms for many women without professional interventions.

Women’s reliance on their family members to the exclusion of health professionals suggests that families provide a socially supportive environment that can be the basis for therapeutic interventions when needed [50]. Most Chinese women still practice doing the month and a small case report found it may reduce women’s risk for depression [51,52]. It suggests that perinatal depression, especially postnatal, could be usefully managed in traditional family settings, making use of the special nature of interactions afforded by the practice of doing the month.

Our findings point to the need and opportunity for the development of culturally attuned ways of addressing the needs of women with symptoms indicative of PND. First, we recommend building on women’s preferences for family support and care and the emphasis in Chinese culture on family bonding and family responsibility for the care of pregnant and postpartum women. Second, we recommend educating women and their families about PND, especially emphasizing when it would be timely to seek professional help if their condition does not improve or deteriorates, considering the importance of the family structure in dealing with PND. Lastly, as 83% of our respondents expressed an interest in trying smartphone-app-based therapies, well-designed pragmatic trials may be useful to assess the effectiveness of those apps in real-world settings.

There are several limitations to the study. The cohort on which this study was based was a convenience sample. Although we approached every eligible woman during the 4-month recruitment period, we did not record their overall number or basic demographic features, and thus were unable to rule out undetected forms of sample bias. Only 64.9% of the high-risk participants completed the questionnaire (161 of 248). As this was an anonymous survey, we do not have information on who completed the questionnaire or whether individuals’ likelihood to respond to the survey was associated with their conditions. Finally, it is possible that the EPDS is overly sensitive to the complaints among Chinese women such that their scores overstate what are generally mild complaints. If this were true, their reticence to seek follow-up consultation may have been a reflection of their relatively unimpaired conditions. However, we sought to set a high bar for positive screening, and we did not find an inordinate proportion of screen-positive respondents when comparing our results with prior reports.

## 5. Conclusions

Depressive symptoms are common among Chinese women during the perinatal period; however, the majority of symptom-positive women do not accept subsequent referral to mental health services. Our result argues against referring all screen-positive women for professional services at this time. Feasible options should build upon the tradition of family support for more engaged and educated response to PND.

## Figures and Tables

**Table 1:** Participant profile (*n* = 1126).

Variable	High Risk Women (*n* = 248)	Other Women in Cohort (*n* = 878)	*p*
Age (years)			0.905
<25	37 (15.04%)	120 (13.89%)	
25–30	139 (56.50%)	480 (55.56%)	
30–35	51 (20.73%)	188 (21.76%)	
>35	19 (7.72%)	76 (8.80%)	
Average	28.28	28.83	
Missing	2	14	
Parity			0.806
Primiparas	64 (25.91%)	229 (26.97%)	
Multiparas	183 (74.09%)	620 (73.03%)	
Missing	1	29	
Education			0.891
Middle school graduate or less	31 (12.60%)	96 (11.03%)	
High school	58 (23.58%)	200 (22.99%)	
College degree	136 (55.28%)	501 (57.59%)	
Graduate or professional degree	21 (8.54%)	73 (8.39%)	
Missing	2	8	
Jobs			0.900
Government workers	51 (21.07%)	185 (21.46%)	
Enterprise managers	43 (17.77%)	151 (17.52%)	
Private business owner	31 (12.81%)	136 (15.78%)	
Workers/Farmers	11 (4.55%)	39 (4.52%)	
Unemployed (due to disease or other reasons)	66 (27.27%)	214 (24.83%)	
Others	40 (16.53%)	137 (15.89%)	
Missing	6	16	
Income per month			0.471
0	69 (28.28%)	236 (27.73%)	
¥1–2000	12 (4.92%)	53 (6.23%)	
¥2001–5000	122 (50.00%)	389 (45.71%)	
Over ¥5001	41 (16.80%)	173 (20.33%)	
Missing	4	27	
Depression history			0.005 **
No	164 (93.18%)	609 (97.91%)	
Yes	12 (6.82%)	13 (2.09%)	
Missing	72	256	
Living Situation			0.233
Nuclear family	87 (35.80%)	375 (43.20%)	
Nuclear family and parents in law	91 (37.45%)	296 (34.10%)	
Nuclear family and parents	40 (16.46%)	107 (12.33%)	
Living alone	20 (8.23%)	71 (8.18%)	
Else	5 (2.06%)	19 (2.19%)	
Missing	5	10	
Relationship with spouse			<0.001 ***
Satisfied	174 (71.90%)	731 (84.12%)	
Somewhat satisfied	65 (26.86%)	136 (15.56%)	
Dissatisfied	2 (1.24%)	2 (0.23%)	
Missing	7	9	
Family’s wishes about child’s sex			0.041 *
Boy	44 (18.11%)	106 (12.24%)	
Girl	32 (13.17%)	103 (11.89%)	
Does not matter	167 (68.72%)	657 (75.87%)	
Missing	5	12	
Willingness to do the month			0.018 *
Yes	207 (85.19%)	786 (90.66%)	
No	36 (14.81%)	81 (9.34%)	
Missing	5	11	

* *p* ≤ 0.05; ** *p* ≤ 0.01; *** *p* ≤ 0.001.

## Appendix A

In the related longitudinal study, we found that even without any medical treatment, many women may be able to recover from their depression. Our web-based survey indicated a large proportion of the high-risk women′s EPDS scores dropped below 12.5, and more than half of them scored below 9.5 at 6 months and one year following the delivery.

**Table A1:** Follow-up about depression of women in the cohort.

EPDS Scores	High-Risk Women (*n* = 248)	Other Women in Cohort (*n* = 878)
**Half year after delivery**		
>12.5	42 (34.43%)	19 (5.85%)
<12.5	80 (65.57%)	306 (94.15%)
missing	126 (50.80%)	553 (63.98%)
**One year after delivery**		
>12.5	26 (23.42%)	11 (3.48%)
<12.5	85 (76.58%)	305 (96.52%)
missing	137 (55.24%)	562 (64.00%)
